# Make or break for mitochondria

**DOI:** 10.7554/eLife.00804

**Published:** 2013-05-14

**Authors:** Catherine L Nezich, Richard J Youle

**Affiliations:** 1 **Catherine L Nezich** is at the Medical Research Council Mitochondrial Biology Unit, Cambridge, United Kingdom and at the National Institute of Neurological Disorders and Stroke, National Institutes of Health, Bethesda, United Statesnezichc@ninds.nih.gov; 2 **Richard J Youle** is an *eLife* reviewing editor, and is at the National Institute of Neurological Disorders and Stroke, National Institutes of Health, Bethesda, United Statesyouler@ninds.nih.gov

**Keywords:** ERMES, Gem1, Miro, mitochondrial DNA, mitochondria, S. cerevisiae

## Abstract

Ensuring that mitochondrial DNA is successfully divided between daughter mitochondria involves a complex series of interactions with the endoplasmic reticulum and a variety of enzymes.

**Related research article** Murley A, Lackner LL, Osman C, West M, Voeltz GK, Walter P, Nunnari J. 2013. ER-associated mitochondrial division links the distribution of mitochondria and mitochondrial DNA in yeast. *eLife* **2**:e00422. doi: 10.7554/eLife.00422

**Image** Mitochondrial division requires the making and breaking of contacts (red regions) between the endoplasmic reticulum (green) and mitochondria (purple)

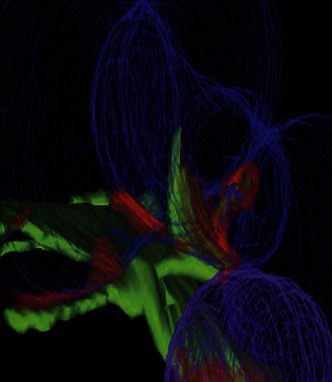

Mitochondria are usually described as being the main source of energy for cells, but they do much more than this. They are, for example, involved in controlling various aspects of the cell cycle and cell growth, and this requires them to undergo repeated cycles of division and fusion. Moreover, mitochondria contain their own DNA, and this needs to be replicated and distributed between the two daughter mitochondria that are produced in the division process.

Mitochondrial division is driven by an enzyme called Dnm1 that assembles into a helix around the mitochondria, and an enzyme called Drp1 performs the same role in mammals. However, the diameter of this helix is much smaller than the diameter of a typical mitochondrion, so the latter must be constricted by something before the helix can form.

It was recently reported that portions of the endoplasmic reticulum (ER) wrap around mitochondria at constriction sites marked by Dnm1/Drp1 helices in yeast/mammalian cells (Friedman et al., 2011). Moreover, depletion of Drp1 does not disrupt the constriction process, which suggests that contact between the ER and mitochondria is a conserved feature of mitochondrial division that precedes the involvement of Dnm1/Drp1.

Contact between the ER and mitochondria enables efficient communication between the two, which is essential for processes such as calcium signaling, lipid biosynthesis and protein import (Rowland and Voeltz, 2012). It is thought that this contact is mediated by one or more protein tethers, but to date only one such tether—a multiprotein complex called the ER-mitochondria encounter structure (ERMES; Kornmann et al., 2009)—has been identified in yeast.

Now, writing in *eLife*, Jodi Nunnari of the University of California at Davis and colleagues–including Andrew Murley as first author–report evidence that ERMES resides at the contact sites between the ER and mitochondria where constriction takes place, and that another enzyme, Gem1, is needed to break these contacts after the mitochondria have divided (Murley et al., 2013). Gem1, which is already known to regulate other aspects of the interaction between the ER and mitochondria (Kornmann et al., 2011), contains two domains that bind calcium ions and two domains that break down GTP molecules (which are closely related to the ATP molecules used by mitochondria to store chemical energy).

Nunnari and co-workers—who are based at UC Davis, UC San Francisco and the University of Colorado at Boulder—used time-lapse confocal microscopy to study fluorescently-tagged ERMES and Gem1 proteins in living yeast cells. The ERMES subunits were found to localize to the constriction sites in both wild-type yeast and in mutant yeast lacking Gem1 (Figure 1). The mitochondria also went on to divide successfully in both types of yeast, but both daughter mitochondria remained stuck to the ER in the mutant yeast cells (Figure 1). This suggests that Gem1 is required to disengage or mobilize ER–mitochondria contacts, which is important for the proper distribution of mitochondria in cells.

**Figure 1.**
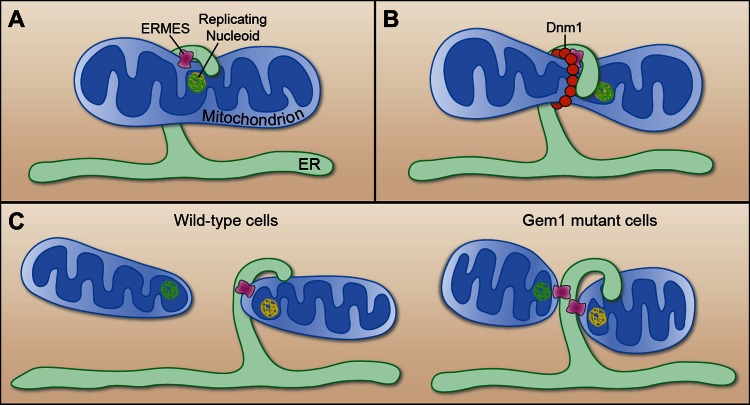
Mitochondrial division. (**A**) Murley et al. demonstrate that the ERMES tethering complex (pink) localizes to the region where the endoplasmic reticulum (ER) makes contact with a mitochondrion in yeast, and where the nucleoid that contains the mitochondrial DNA is replicating. (**B**) The mitochondrion undergoes constriction at this contact site, which allows a helix of Dnm1 (red) to form around it. (**C**) This helix leads to further constriction and, ultimately, to the division of the mitochondrion and the formation of two daughter mitochondria in wild-type cells (left). Each daughter mitochondrion has its own nucleoid. Significantly, one daughter remains attached to the ER via ERMES, which remains intact through the division process. However, in cells lacking Gem1, both of the daughter mitochondria remain tethered to the same ER segment, and both have somewhat unusual shapes (right).

FIGURE CREDIT: CATHERINE L NEZICH

Murley et al. also added fluorescent tags to nucleoids—the structures that contain the mitochondrial DNA—and confirmed that they were located adjacent to ERMES complexes (Hobbs et al., 2001) at the majority of the constriction/division sites. Before division occurred these nucleoids were seen to exhibit behaviour that is indicative of DNA replication taking place, and after division they were mostly observed at the tips of both of the new daughter mitochondria. The contact sites between the ER and mitochondria thus appear to preferentially form near replicating nucleoids in order to assist with the distribution of DNA between the daughter mitochondria (Figure 1).

But how does Gem1 function? Murley et al. demonstrate that the GTPase activity of this protein, but not its calcium-binding activity, is required for the final release of ER–mitochondria contacts, which is consistent with the results of mitochondrial inheritance studies (Koshiba et al., 2011). It has been suggested that ERMES components link the nucleoids to the cellular cytoskeleton (Boldogh et al., 2003). This notion is compatible with the results of experiments which found that the enzymes Miro1 and 2—which are similar to Gem1 in many ways—mobilize mitochondria in mammalian cells by interacting with components of the cytoskeleton (Glater et al., 2006). Gem1 could thus be involved in the recruitment of protein motility factors that promote the spatial separation of the daughter mitochondria following the division process. Alternatively, Gem1 might act directly on ERMES to regulate the physical link between the ER and mitochondria. Consistent with this idea, Miro1 localizes to ER-mitochondria contact sites in mammalian cells, and Gem1 associates with ERMES subunits and regulates the number and size of ERMES foci in yeast (Kornmann et al., 2011).

The discovery of a novel role for ERMES and Gem1 proteins by Murley et al. raises new questions about mitochondrial division. First, does the ER facilitate constriction and, if so, how? One possibility is that an exchange of lipids at the ER–mitochondria interface alters the content of the mitochondrial membrane in this specific region, and that this leads to the constriction required for Dnm1 helix formation. Alternatively, ERMES (or its as yet unidentified mammalian equivalent) may facilitate the generation of a constricting force via actin polymerization (Korobova et al., 2013). And how is Dnm1 recruited to the constriction? Possibilities include via the ERMES complex itself or via some component of the ER that we don’t yet know about. Or maybe the curvature of the membrane activates the proteins that help to recruit the Dnm1/Drp1 enzymes that form the helix around the constriction.

While the present results leave these and many other questions to be resolved, future research delving into these unknowns should lead to a better understanding of the many neurodegenerative diseases that result from errors in the process of mitochondrial division.
